# Comparison of the depolarization response of human mesenchymal stem cells from different donors

**DOI:** 10.1038/srep18279

**Published:** 2015-12-14

**Authors:** Sarah Sundelacruz, Michael Levin, David L. Kaplan

**Affiliations:** 1 Department of Biomedical Engineering, Tufts University, Medford, MA, USA; 2 Department of Biology, and Center for Regenerative and Developmental Biology, Tufts University, Medford, MA, USA

## Abstract

Bioelectric signaling is currently being explored as a novel regulator of cell processes in non-excitable cells. In particular, stem cells have demonstrated increasing evidence of electrophysiology-mediated regulation of stemness acquisition, proliferation, differentiation, and migration. However, in light of many reports of primary stem cell heterogeneity, it is important to characterize the variability of stem cell response to biophysical manipulations in order to assess the utility of bioelectric modulation as a universal strategy for stem cell control. In this work, human mesenchymal stem cells (hMSCs) from five donors were evaluated for their response to membrane potential (V_mem_) depolarization. We compared the inter-donor variability of their osteogenic and adipogenic differentiation potential, as well as their ability to maintain a differentiated phenotype after induction. We identified the markers that responded most consistently across donors and found that calcium deposition and gene expression of bone sialoprotein, lipoprotein lipase, and fatty acid binding protein 4 are the preferred markers for assessing differentiation response to V_mem_ depolarization. We also note that since there exists variability even among some of these markers, these assays should be performed on any newly acquired hMSC population if their bioelectric properties are to be studied further.

Bone marrow-derived human mesenchymal stem cells (hMSCs), the non-hematopoietic, self-renewing, stromal cell fraction of the bone marrow^1^,^2^, are an attractive cell source for stem cell therapies and for regenerative medicine^3^,^4^,^5^. There exists a large body of work characterizing hMSC response to a range of biophysical and biochemical stimuli, including growth factors, cytokines, extracellular matrices, mechanical stimuli, oxygen tension, and extrinsic and intrinsic electrical fields^6^,^7^,^8^,^9^,^10^. A major goal of these characterization studies is to identify optimal stimuli for achieving desired cell functions, such as maintenance of stemness, proliferation, differentiation, and migration.

HMSCs are promising candidates for cell transplantation therapies: they have been exploited for tissue repair and regeneration in orthopedic, hematopoietic, cardiac, gastrointestinal, pancreatic, renal, kidney, pulmonary, hepatic, and neurological tissues, as well as for their immunomodulatory and anti-inflammatory properties^11^,^12^,^13^,^14^. However, pre-clinical and clinical trial data point to the need for better optimization of MSC-based therapies^3^,^4^,^15^,^16^. One factor that may hamper this optimization is the heterogeneity of hMSC properties depending on the donor source of the primary cells. In order for hMSCs to be a reliable cell source for therapeutic applications, it is therefore necessary to understand how cell behavior differs between donors. Studies comparing bone marrow mesenchymal stem cells or stromal cells from multiple donors have found significant differences in cell growth rate^17^, alkaline phosphatase expression and activity^17^,^18^, osteogenic gene expression^17^,^18^, mineralization^18^, bone-forming capacity^18^,^19^, adipocyte precursor frequency^20^, surface marker expression^20^,^21^, global proteomic signature^22^, and endothelial-like tube formation^23^. Variables such as donor age and gender affect the function of hMSCs^21^,^24^. Thus, it is clear from the literature that the inherent properties of hMSCs can vary significantly. If native hMSCs demonstrate such variability, it is possible that their response to experimental manipulation may also vary. Studies of the response of primary stem cells to applied biophysical or biochemical stimuli should therefore examine the behavior of cells from multiple donors in order to obtain a better understanding of the inter-donor heterogeneity of the observed response.

Bioelectric signaling is one aspect of stem cell biology that is only beginning to be studied and understood. Bioelectric signaling is known to play a role in a wide range of cell functions, including cell proliferation, migration, differentiation, wound healing, pattern formation, and tissue regeneration^25^,^26^,^27^,^28^,^29^,^30^,^31^,^32^. The role of intrinsic cell electrophysiology in the regulation of cell function is an area of active investigation for non-excitable cells, including stem cells. HMSCs express several ion currents, including Ca^2+^-dependent K^+^ currents, delayed rectifier K^+^ currents, transient outward K^+^ currents, and slow-activating currents^33^. They express several ion channels, including Kv4.3, MaxiK, L-type Ca^2+^ channels, and hyperpolarization-activated cyclic nucleotide-gated (HCN) ion channels^34^. However, the expression of these ion channels and currents has not been investigated in terms of consistency of response among primary cells from different donors. Beyond its relevance for potential excitable functions, hMSC electrophysiology is also of interest because of its role in regulating hMSC differentiation toward osteogenic (OS) and adipogenic (AD) lineages. We have previously reported that the resting membrane potential (V_mem_) of hMSCs undergoes endogenous hyperpolarization during both OS and AD differentiation, and that this hyperpolarization is required for differentiation^35^. Artificial depolarization suppresses OS and AD differentiation, while artificial hyperpolarization augments OS differentiation^35^. Endogenous V_mem_ also plays a role in maintenance of the differentiated phenotype in hMSCs that have been pre-differentiated toward osteoblasts or adipocytes^36^. The ability to control cell fate and lineage decisions using electrophysiology would be a powerful addition to current methods of stem cell regulation. However, to date, efforts toward this end have not included a systematic assessment of the heterogeneity of hMSC response to bioelectric signals, which is critical for understanding how effective this strategy will be for primary cells that may differ significantly from donor to donor. More broadly, an understanding of robustness and intrinsic variability in bioelectric network pathways is important in order to take advantage of voltage-based pathways for regenerative medicine and synthetic bioengineering applications^37^,^38^,^39^,^40^,^41^,^42^.

In the present study, we examined the effects of V_mem_ depolarization on the differentiation response of hMSCs derived from five different donors. First, we depolarized hMSCs undergoing OS or AD differentiation to investigate the effects of depolarization on the differentiation process. Subsequently, we depolarized hMSCs after pre-differentiating them toward osteoblasts or adipocytes to investigate the effects of depolarization on maintenance of the differentiated phenotype. We compared the five cell sources to determine whether the hMSCs exhibited a consistent response to electrophysiological modulation. We also compared different tissue-specific markers to determine which markers displayed the most consistent response to depolarization. To our knowledge, this is the first report addressing the inter-donor heterogeneity of stem cell responses to electrophysiological modulation.

## Materials and Methods

### Cell culture

Bone marrow aspirates were obtained through Lonza’s Research Bone Marrow Donor Program (Hopkinton, MA) following approved guidelines for informed consent. Bone marrow donors were healthy males between the ages of 18 to 25 (Donor A, 25 yrs; Donor B, 25 yrs; Donor C, 22 yrs; Donor D, 25 yrs; Donor E, 18 yrs). HMSCs were isolated from aspirates as previously described^35^. Briefly, whole bone marrow aspirates were plated in tissue-culture treated flasks at a density of 10 uL aspirate per cm^2^ and were cultured in control medium (Dulbecco’s Modified Eagle Medium (DMEM) with 10% fetal bovine serum, penicillin (100 U/mL), streptomycin (100 μg/mL), and 0.1 mM non-essential amino acids) supplemented with basic fibroblast growth factor (bFGF, 1 ng/mL) (Invitrogen, Carlsbad, CA). Cells were cultured in a humidified incubator at 37 °C with 5% CO_2_. Hematopoietic stem cells suspended in the aspirate were removed from the adherent hMSCs after approximately five days. Upon reaching near confluence, cells were trypsinized and frozen. For subsequent cell expansion, cells were grown in control medium at 37 °C maintained at 5% CO_2_ and 5% O_2_. To begin differentiation, cells (passages two through four) were switched to either osteogenic or adipogenic differentiation media and normoxic conditions. Osteogenic medium consisted of α-MEM supplemented with 10% FBS, penicillin (100 U/mL), streptomycin (100 μg/mL), 10 mM β-glycerophosphate, 0.05 mM L-ascorbic acid-2-phosphate, and 100 nM dexamethasone (Sigma-Aldrich, St. Louis, MO). Adipogenic medium consisted of control medium supplemented with 0.5 mM 3-isobutyl-1-methyl-xanthine, 1 μM dexamethasone, 5 μg/mL insulin, and 50 μM indomethacin (Sigma-Aldrich, St. Louis, MO). Undifferentiated hMSCs were maintained in control medium.

### V_mem_ depolarization

To induce V_mem_ depolarization, the concentration of K^+^ in the extracellular medium was elevated by the addition of potassium gluconate to the differentiation media. Changing the extracellular [K^+^] is predicted to alter V_mem_ according to the Goldman-Hodgkin-Katz equation. V_mem_ depolarization in hMSCs in response to elevated extracellular [K^+^] has been previously measured with sharp microelectrode recordings and with voltage-sensitive dyes^35^. The final concentration of supplemented [K^+^] was 40 mM for osteogenic differentiation studies and 80 mM for adipogenic differentiation studies. For studies of the effect of depolarization on osteogenic or adipogenic differentiation, depolarization and differentiation were started on the same day, and this was considered Day 0. Depolarizing medium was replenished at each medium change until cells were harvested for the various time point analyses.

For the second set of studies of the effect of depolarization after pre-differentiation, the cells were first pre-treated for two weeks in differentiation or control medium. After this period of pre-treatment, cells were switched to either high K^+^- containing differentiation medium, normal differentiation medium, or control medium for one additional week before harvesting cells for analysis. All analyses for this second set of studies were performed at this time point.

### Quantitative PCR

Quantitative PCR was performed to evaluate gene expression in depolarized cells. For the studies of depolarization during differentiation, RNA was collected on Day 7, while for the studies of depolarization after pre-differentiation, RNA was collected on Day 21. RNA was isolated by phenol-chloroform extraction, followed by additional purification using RNEasy mini spin columns (Qiagen). Reverse transcription was performed using the High-Capacity cDNA Archive Kit following the manufacturer’s instructions (Applied Biosystems, Foster City, CA). Osteogenic and adipogenic gene transcripts were amplified using the Brilliant III Fast qPCR Master Mix (Agilent Technologies) and quantified on a Stratagene Mx3000P QPCR System (Stratagene, La Jolla, CA). Primers and probes for osteogenic and adipogenic genes were obtained from TaqMan® Gene Expression Assay kits (Applied Biosystems). Transcript levels were normalized to the housekeeping gene glyceraldehyde 3-phosphate dehydrogenase (GAPDH) and were reported relative to corresponding levels in undifferentiated hMSCs (n = 3–6).

### Alkaline phosphatase (ALPL) activity

ALPL activity was measured on Day 14 for cells depolarized during osteogenic differentiation and on Day 21 for cells depolarized after pre-differentiation. ALPL activity was quantified in osteogenic cells using a biochemical assay that measures the amount of p-nitrophenol converted from p-nitrophenyl phosphate (pNPP) by ALPL. Samples were lysed in 0.2% v/v Triton X-100 in 5 mM MgCl_2_, centrifuged at 16,000g at 4 °C for 10 min, and cleared of cellular debris. Supernatants were incubated at 37 °C with pNPP substrate in 2-amino-2-methyl-1-propanol buffer for 1.5 hours. The reaction was stopped with 0.2 M NaOH, and the absorbance of the colored product was measured at 405 nm (n = 3–8).

### Calcium quantification

Total calcium levels were quantified on Day 21 for all osteogenic samples using the Calcium CPC Liquicolor Kit (Stanbio Laboratory) according to the manufacturer’s instructions. Calcium was eluted from samples by incubation in 5% trichloroacetic acid. Cellular debris was removed by centrifugation at 16000g for 5 min at 25 °C. Samples were incubated with assay reagent for 5 min, and the absorbance of the end product was measured at 575 nm (n = 3–7).

### DNA quantification

For normalization of ALP activity and calcium quantification data on Days 14 and 21, DNA content of samples was measured using the Quant-iT PicoGreen dsDNA Assay Kit (Invitrogen) according to the manufacturer’s instructions. Briefly, samples were lysed in 0.2% v/v Triton X-100 in 5 mM MgCl_2_, centrifuged at 16000g at 4 °C for 10 min, and cleared of cellular debris. Supernatants were diluted with TE buffer and incubated with PicoGreen reagent, and fluorescence was measured at excitation and emission wavelengths of 480 and 520 nm, respectively (n = 3–7).

### Oil Red O (ORO) staining

Lipid droplets were stained with ORO and visualized by light microscopy on Day 21 for adipogenic samples. ORO stock solution was prepared by dissolving 40 g ORO powder into 200 mL isopropanol. A working solution of ORO was prepared by mixing 6 parts ORO stock solution with 4 parts isopropanol. Cells were fixed with 10% formalin, then stained with working ORO solution for 30 min. The staining solution was removed and the cells were washed 4 times with distilled water. Cells were imaged with an inverted microscope (Axiovert S100, Carl Zeiss, Inc.) equipped with Zeiss A-Plan 10× (NA 0.25) and LD A-Plan 32× (0.40) objectives. Images captured by a Sony Exwave HAD CCD camera were acquired using ImageJ software (NIH). Photoshop software (Adobe Systems Inc.) was used to adjust brightness and contrast levels over the entire image.

### Statistics

Data are reported as mean ± standard deviation. One-way ANOVA was performed, followed by a Tukey-Kramer post-hoc test. Differences between data points were considered statistically significant if p < 0.05. In bar graphs, groups that are statistically different (p < 0.05) are labeled with different letters.

## Results

### Depolarization during osteogenic differentiation

**Figure 1:**
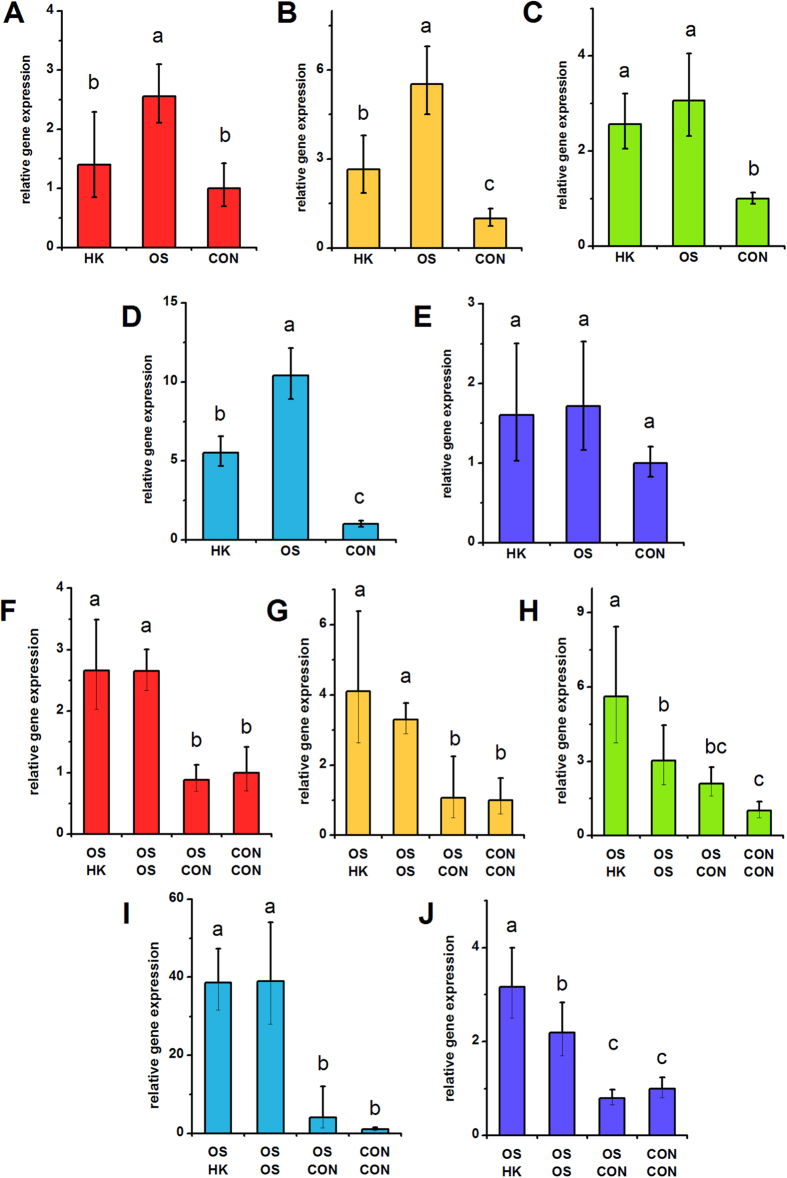
***ALPL*** **expression in hMSCs depolarized during osteogenic differentiation (A–E) and in pre-differentiated hMSCs (F–J).** (**A**–**E**) *ALPL* gene expression was quantified by qPCR after 7 days of hMSC culture in osteogenic medium +40 mM K^+^ (HK), osteogenic medium (OS), or control medium (CON). (**F**–**J**) hMSCs were pre-treated for 2 weeks (OS or CON medium) before switching to HK, OS, or CON medium for 1 week. *ALPL* gene expression was quantified by qPCR after the entire 3 weeks of culture. Data points are mean relative expression ±standard deviation, n = 3–6. Panels **A**,**F**; **B**,**G**; **C**,**H**; **D**,**I**; and **E**,**J** correspond to data from Donors A–E, respectively. Different letters over bar graphs represent statistically different groups as determined by one-way ANOVA and the Tukey-Kramer post-hoc test, p < 0.05.

**Figure 2:**
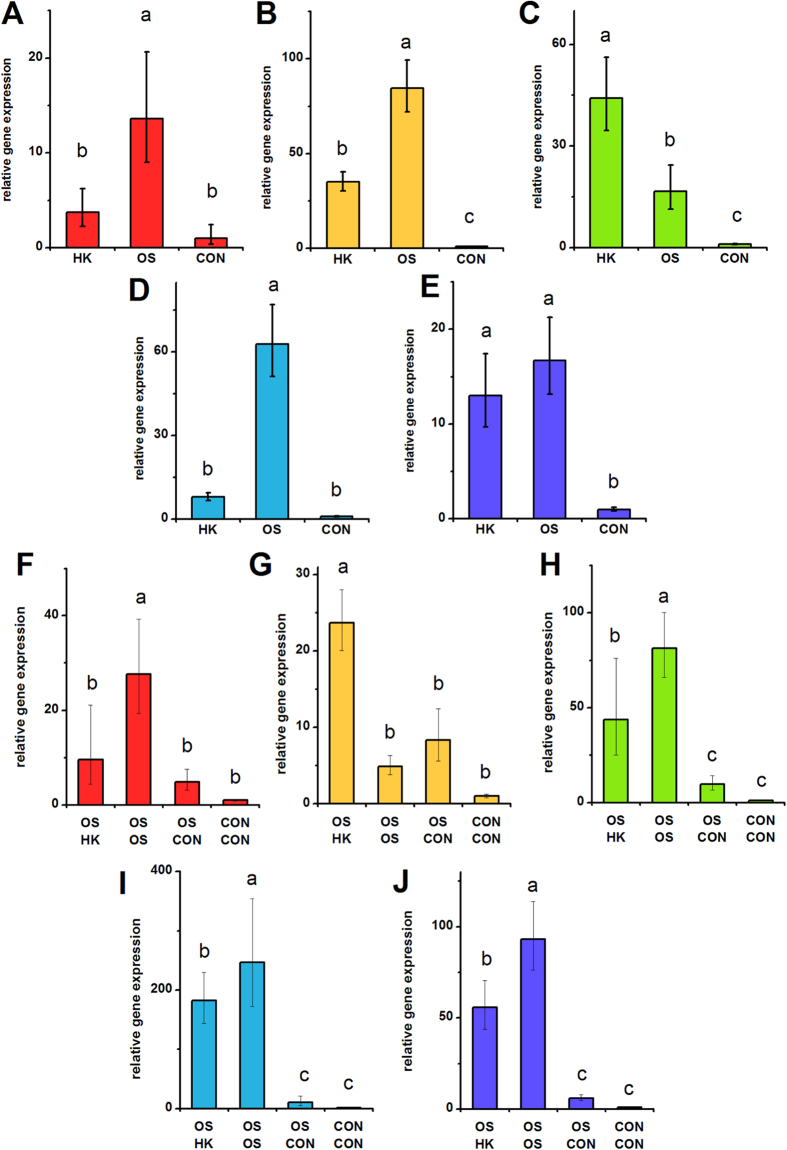
***IBSP*** **expression in hMSCs depolarized during osteogenic differentiation (A**–**E) and in pre-differentiated hMSCs (F**–**J).** (**A**–**E**) *IBSP* gene expression was quantified by qPCR after 7 days of hMSC culture in osteogenic medium +40 mM K^+^ (HK), osteogenic medium (OS), or control medium (CON). (**F**–**J**) hMSCs were pre-treated for 2 weeks (OS or CON medium) before switching to HK, OS, or CON medium for 1 week. *IBSP* gene expression was quantified by qPCR after the entire 3 weeks of culture. Data points are mean relative expression ±standard deviation, n = 3-6. Panels **A**,**F**; **B**,**G**; **C**,**H**; **D**,**I**; and **E**,**J** correspond to data from Donors **A**–**E**, respectively. Different letters over bar graphs represent statistically different groups as determined by one-way ANOVA and the Tukey-Kramer post-hoc test, p < 0.05.

**Figure 3:**
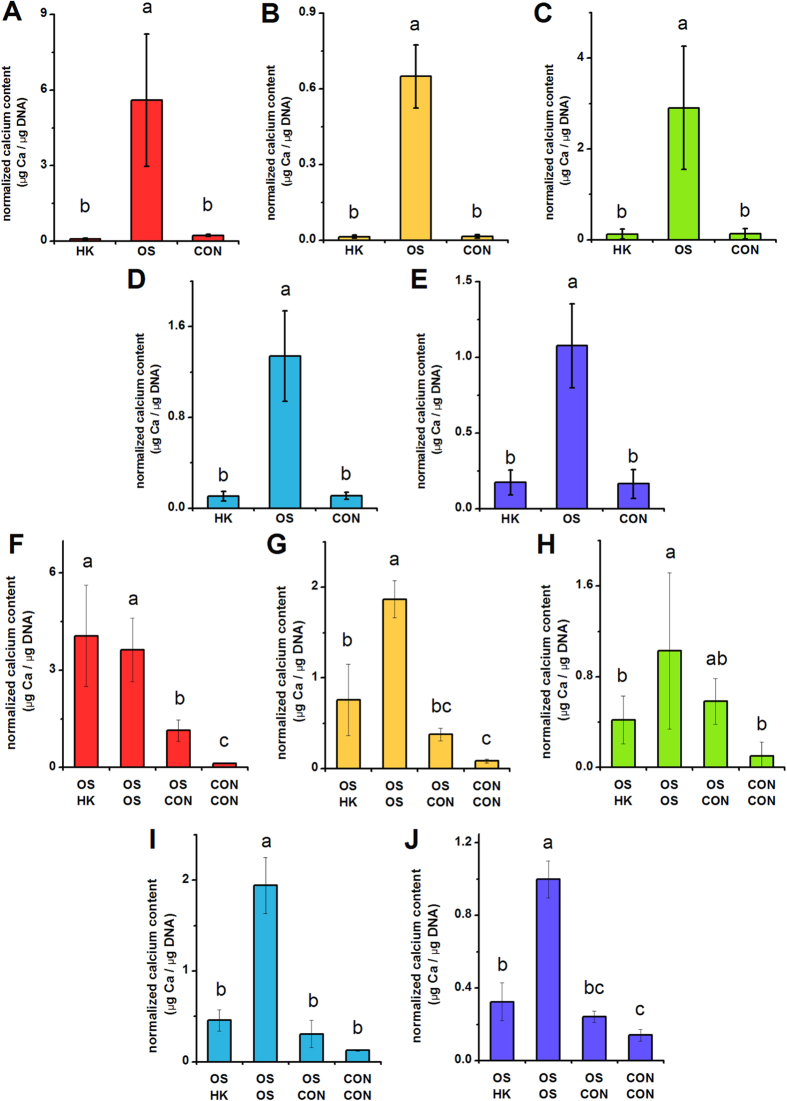
**Total calcium content of hMSCs depolarized during osteogenic differentiation (A**–**E) and in pre-differentiated hMSCs (F**–**J).** (**A**–**E**) Total calcium content was quantified by a biochemical assay after 21 days of hMSC culture in osteogenic medium +40 mM K^+^ (HK), osteogenic medium (OS), or control medium (CON). (**F**–**J**) hMSCs were pre-treated for 2 weeks (OS or CON medium) before switching to HK, OS, CON for 1 week. Total calcium content was quantified after the entire 3 weeks of culture. Data points represent mean normalized calcium content (μg Ca per μg DNA) ±standard deviation, n = 3–7. Panels **A**,**F**; **B**,**G**; **C**,**H**; **D**,**I**; and **E**,**J** correspond to data from Donors **A**–**E**, respectively. Different letters over bar graphs represent statistically different groups as determined by one-way ANOVA and the Tukey-Kramer post-hoc test, p < 0.05.

We quantified several bone markers to determine whether depolarization affects the progression of osteogenic differentiation: gene expression of alkaline phosphatase (ALPL) and bone sialoprotein (IBSP) on Day 7; ALPL activity on Day 14, and calcification on Day 21. ALPL expression was lowered in HK-treated cells of Donors A, B, and D by 1.75-, 1.98-, and 1.88-fold, respectively (Fig. 1A–E, p < 0.05). The two remaining donors did not exhibit a statistical difference in ALPL expression; one of these donors additionally did not exhibit a difference in ALPL levels between the osteogenic and undifferentiated groups. IBSP expression was consistently and significantly increased upon osteogenic differentiation among all donors (Fig. 2A–E). IBSP levels were lowered in HK-treated cells of Donors A, B, and D, showing a 3.32-, 2.41-, and 7.88-fold difference (p < 0.05). IBSP levels did not significantly response to HK in Donor E, while IBSP levels unexpectedly increased in HK-treated Donor C cells (2.63-fold, p < 0.001). ALPL activity, measured on Day 14, was an overall poor indicator of osteogenic differentiation. Only one donor (Donor D) exhibited a significant increase in ALPL activity after osteogenic differentiation relative to undifferentiated cells, and the effect of HK on the enzyme activity varied widely among donors, including increased activity, decreased activity, and no effect relative to the osteogenic group (Supplemental Fig. 1A–E). Total calcium content, by contrast, was a reliable osteogenic marker: calcium content was elevated between 6.56- to 24.18-fold in all osteogenic cells compared to undifferentiated cells (Fig. 3A–E, p < 0.01). The effect of HK on calcium content was similarly consistent, decreasing significantly in all donor cells (Fig. 3A–E, 6.21- to 62.94-fold, p < 0.01). Overall, we conclude that IBSP expression and calcification are the most consistent osteogenic markers for donor-to-donor comparison of hMSC responses to V_mem_ perturbation, and that HK suppression of these markers is seen for three out of five donors and five out of five donors, respectively. ALP expression and activity, in contrast, were less consistent among donors and therefore may not be suitable markers for evaluating differentiation response.

### Depolarization during adipogenic differentiation

**Figure 4:**
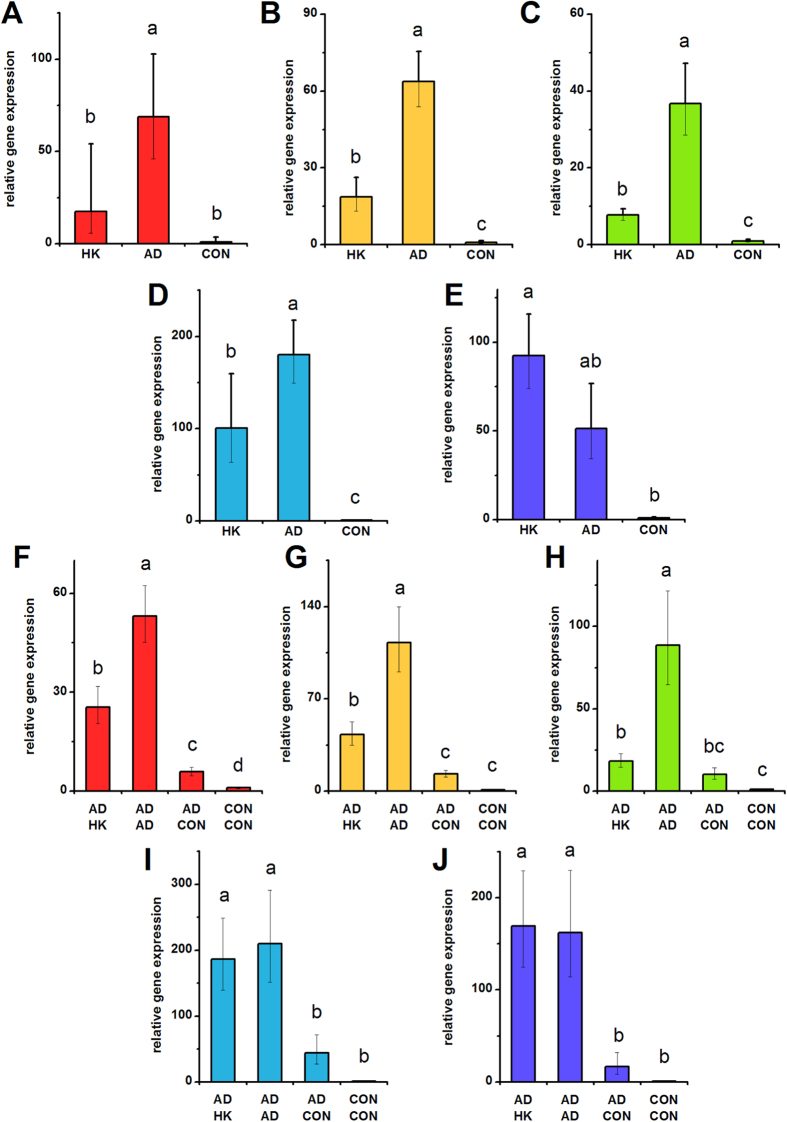
***PPARG*** **expression in hMSCs depolarized during adipogenic differentiation (A**–**E) and in pre-differentiated hMSCs (F**–**J).** (**A**–**E**) *PPARG* gene expression was quantified by qPCR after 7 days of hMSC culture in adipogenic medium +80 mM K^+^ (HK), adipogenic medium (AD), or control medium (CON). (**F**–**J**) hMSCs were pre-treated for 2 weeks (AD or CON medium) before switching to HK, AD, or CON medium for 1 week. *PPARG* gene expression was quantified by qPCR after the entire 3 weeks of culture. Data points are mean relative expression ±standard deviation, n = 4–6. Panels **A**,**F**; **B**,**G**; **C**,**H**; **D**,**I**; and **E**,**J** correspond to data from Donors A–E, respectively. Different letters over bar graphs represent statistically different groups as determined by one-way ANOVA and the Tukey-Kramer post-hoc test, p < 0.05.

**Figure 5:**
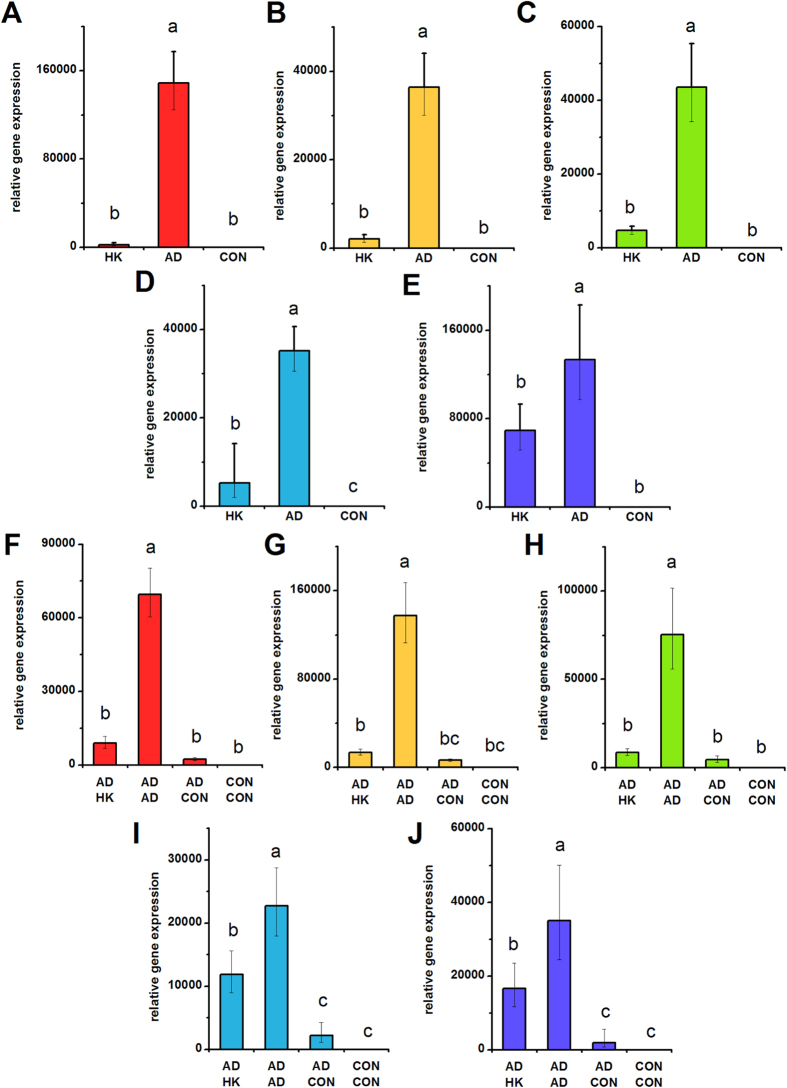
***LPL*** **expression in hMSCs depolarized during adipogenic differentiation (A**–**E) and in pre-differentiated hMSC (F**–**J).** (**A**–**E**) *LPL* gene expression was quantified by qPCR after 7 days of hMSC culture in adipogenic medium + 80 mM K^+^ (HK), adipogenic medium (AD), or control medium (CON). (**F**–**J**) hMSCs were pre-treated for 2 weeks (AD or CON medium) before switching to HK, AD, or CON medium for 1 week. *LPL* gene expression was quantified by qPCR after the entire 3 weeks of culture. Data points are mean relative expression ±standard deviation, n = 4–6. Panels **A**,**F**; **B**,**G**; **C**,**H**; **D**,**I**; and **E**,**J** correspond to data from Donors A–E, respectively. Different letters over bar graphs represent statistically different groups as determined by one-way ANOVA and the Tukey-Kramer post-hoc test, p < 0.05.

**Figure 6:**
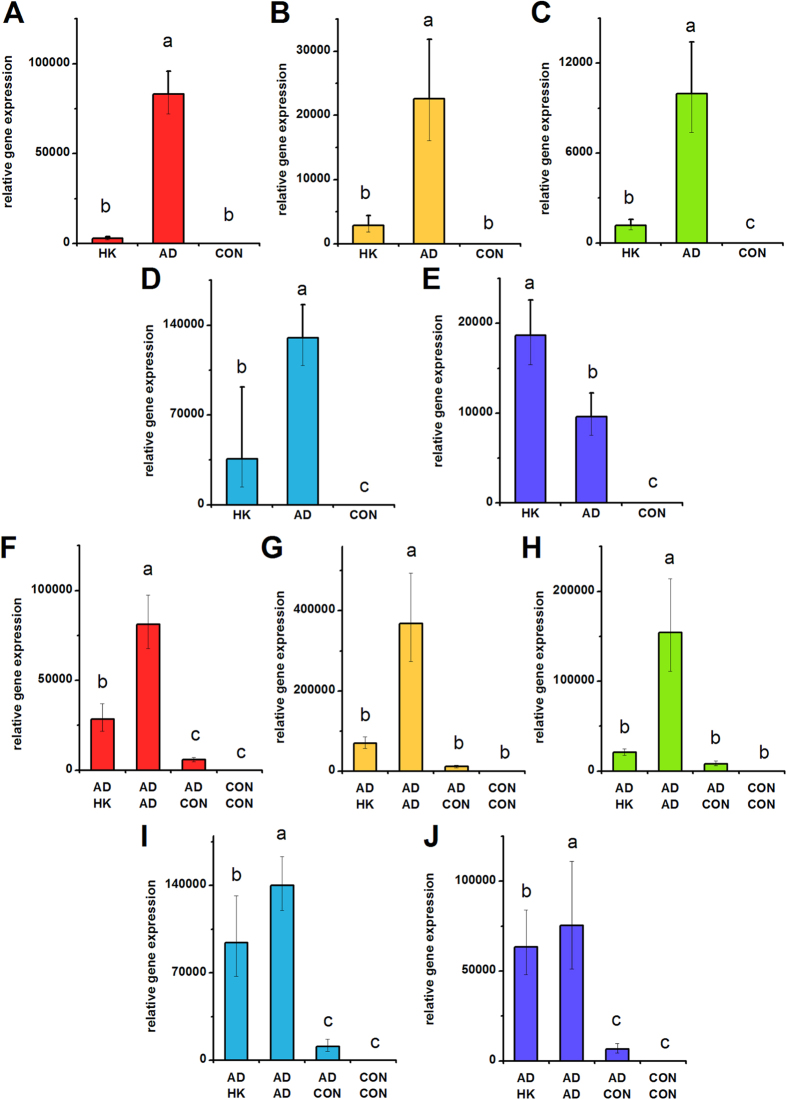
***FABP4*** **expression in hMSCs depolarized during adipogenic differentiation (A**–**E) and in pre-differentiated hMSCs (F**–**J).** (**A**–**E**) *FABP4* gene expression was quantified by qPCR after 7 days of hMSC culture in adipogenic medium +80 mM K^+^ (HK), adipogenic medium (AD), or control medium (CON). (**F**–**J**) hMSCs were pre-treated for 2 weeks (AD or CON medium) before switching HK, AD, or CON medium for 1 week. *FABP4* gene expression was quantified by qPCR after the entire 3 weeks of culture. Data points are mean relative expression ±standard deviation, n = 4–6. Panels **A**,**F**; **B**,**G**; **C**,**H**; **D**,**I**; and **E**,**J** correspond to data from Donors A–E, respectively. Different letters over bar graphs represent statistically different groups as determined by one-way ANOVA and the Tukey-Kramer post-hoc test, p < 0.05.

**Figure 7:**
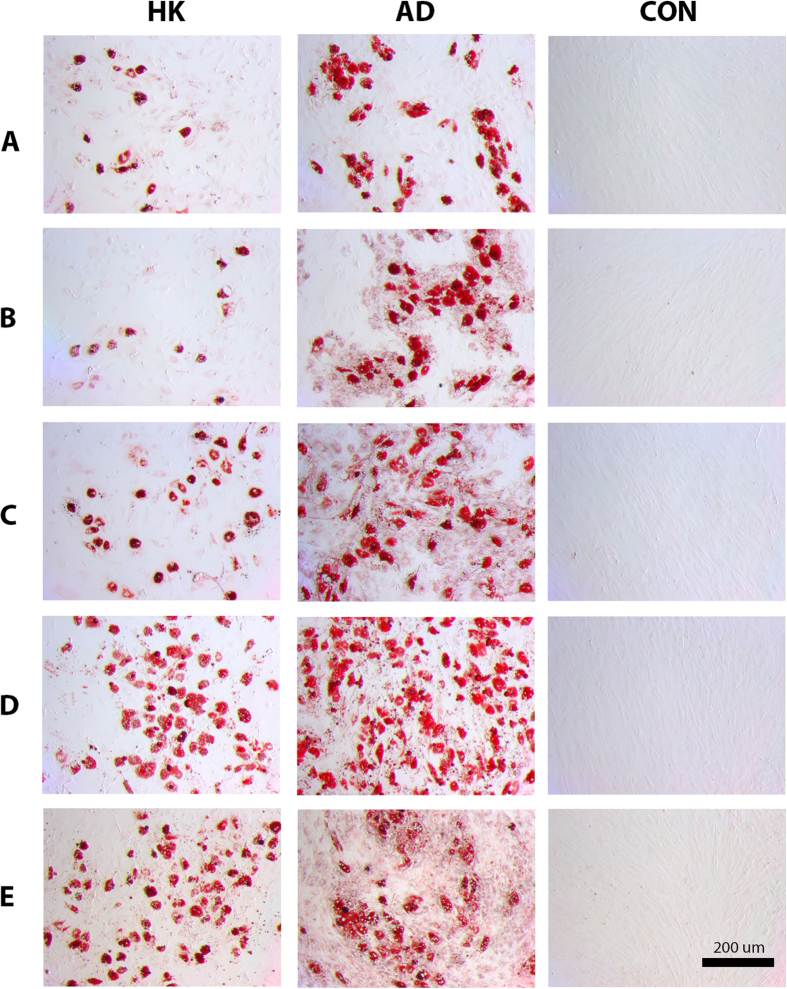
Oil Red O staining of lipids in hMSCs depolarized during adipogenic differentiation. Oil Red O staining of lipid droplets in hMSCs cultured for three weeks in adipogenic medium +80 mM K^+^ (HK), adipogenic medium (AD), or control medium (CON). Rows **A**–**E** correspond to images from Donors A–E, respectively. Scale bar = 200 μm.

HMSCs were differentiated into adipocytes and evaluated for the effect of HK treatment on several adipogenic markers, including peroxisome proliferator-activated receptor γ (PPARG), lipoprotein lipase (LPL), and fatty acid binding protein 4 (FABP4) transcript expression (Figs 4, 5, 6A–E). In general, transcripts showed clear upregulation upon adipogenic differentiation compared to undifferentiated hMSCs, establishing their suitability as adipogenic markers. The one exception was PPARG expression in Donor E, where although the transcript was upregulated upon differentiation, the difference was not found to be statistically significant (Fig. 4E, p = 0.066). LPL response to HK treatment was the most consistent among all five donors, exhibiting a decrease in all donors compared to the untreated adipogenic group (Fig. 5A–E, 2.04- to 52.41-fold, p < 0.05). PPARG and FABP4 expression for Donors A, B, C, and D also decreased with HK treatment (Figs 4 and 6A–D, 1.69- to 4.71-fold for PPARG, p < 0.001; and Fig. 7, 2.80- to 26.64-fold for FABP4, p < 0.01). However, in Donor E, PPARG and FABP4 expression increased by 1.74- and 2.08-fold, respectively, with only FABP4 being statistically significant (Figs 4E and 6E, p < 0.01). We conclude that HK-mediated suppression of adipogenic transcript expression is clearly seen in four out of five donors.

Similarly, Oil Red O staining of lipid droplets showed modulation of adipogenic differentiation with depolarization. In Donors A, B, C, and D, HK-depolarized cells clearly exhibited a decreased frequency of positively stained cells (Fig. 7). Donor E cells, however, did not show an appreciable difference between depolarized and non-depolarized adipogenic cells (Fig. 7E). This result is consistent with Donor E cells demonstrating an atypical response to depolarization with respect to PPARG and FABP4 gene expression, as discussed above. Thus, overall, differential Oil Red O staining was seen in four out of five donors, and the one unresponsive donor could be predicted from its aberrant adipogenic gene expression pattern.

### Depolarization after osteogenic pre-differentiation

We conducted similar experiments on hMSCs pre-differentiated into osteoblasts. HK was added to the cells after pre-differentiation, and we measured ALPL and IBSP expression, ALP activity, and calcification on Day 21. ALPL expression, although significantly different in the osteogenic group (OS-OS) relative to the undifferentiated group (CON-CON) in all donors, was either unchanged after HK treatment (Donors A, B, D) or slightly elevated after HK treatment (Donor C: 1.90-fold, p < 0.001; Donor E: 1.44-fold, p < 0.001) compared to the untreated osteogenic group (Fig. 1F–J). As in the above results with hMSCs depolarized immediately at the start of osteogenic differentiation, we found ALPL activity to again be an inconsistent osteogenic marker: osteogenic cells were found to have the same, increased, or decreased enzyme activity relative to undifferentiated cells in the five donors, and depolarization generally had no significant effect in four out of the five donors (Donors B, C, D, E) (Supplemental Fig. 1F–J). IBSP expression was significantly elevated in the osteogenic group compared to the undifferentiated group in four donors (Donors A, C, D, and E) (Fig. 2F–J). Upon HK treatment, these four donors exhibited consistently lower IBSP transcript levels compared to the untreated osteogenic group (1.36- to 2.57-fold decrease, p < 0.05, Fig. 2F–J). Calcification again performed well as a bone marker across donors: in all five donors, osteogenic cells exhibited significantly increased calcium levels relative to undifferentiated cells (7.06- to 31.88-fold increases, p <  < 0.001, Fig. 3F–J). Additionally, HK treatment resulted in significantly decreased calcium levels in four out of five donors (2.46- to 4.25-fold decrease, p < 0.05, Donors B, C, D, E) (Fig. 3F–J). Overall, we found that IBSP transcript levels and calcium levels were the most consistent markers for evaluating maintenance of the osteogenic phenotype, and that depolarization resulted in decreased marker expression in four out of the five donors. Our results were less consistent for ALPL: enzyme activity varied widely among all samples from all donors, such that a definitive pattern was not observed, and transcript expression, while showing consistent upregulation in the osteogenic groups, did not show a clear depolarization response.

### Depolarization after adipogenic pre-differentiation

In hMSCs pre-differentiated toward adipocytes, we evaluated the effect of HK treatment on maintenance of the adipogenic phenotype, as measured by PPARG, LPL, and FABP4 transcript expression on Day 7. We verified that expression of these genes was higher in untreated differentiated cells compared to controls in all donor cells. We then examined the response of cells to one week of HK treatment after two weeks of pre-differentiation. Depolarization suppressed PPARG expression in three out of five donors (Donors A, B, C; 2.1- to 4.9-fold; p < 0.001), while having no significant effect for the other two donors (Donors D and E) (Fig. 4F–J). LPL and FABP4 expression were suppressed by depolarization in all five donors (LPL: 1.9- to 10.1-fold, p < 0.001, Fig. 5F–J; FABP4: 1.2- to 7.4-fold, p < 0.01, Fig. 6F–J). Thus, LPL and FABP4 expression in hMSC-derived adipocytes exhibited a more consistent response to depolarization across donors compared to PPARG expression. However, the extent to which LPL and FABP4 expression was modulated by depolarization was different among donors, with Donors D and E showing the lowest fold-change for these two markers (1.2- to 2.3-fold for Donors D and E, compared to 2.8- to 10.1-fold for Donors A, B, and C).

Oil Red O staining was also performed to visualize lipid formation. Donors A, B, and C exhibited a lower frequency of positively-stained cells in the depolarized group (AD-HK) compared to the adipogenic group (AD-AD), which is in good agreement with the gene expression data (Supplemental Fig. 2A–C). However, Donors D and E did not exhibit an appreciable difference in staining with depolarization (Fig. 2D,E), which is also consistent with the gene expression data that indicated a lower degree of response in these cell lines.

## Discussion

Growing interest in the electrophysiology of pluripotent stem cells has been stimulated by increasing evidence that endogenous bioelectric properties play important roles in non-excitable cell functions, and that these properties may be exploited to control stem cell behavior and better characterize them for therapeutic purposes. For example, the transcriptome of induced pluripotent stem (iPS) cells has been profiled, revealing significant expression of Ca_v_, Na_v_, Kv, KCa, 2-pore K^+^, and HCN channels. iPS cells expressed IK_DR_ currents, and K^+^ channel block with tetraethylammonium (TEA) inhibited proliferation and viability, reduced the proportion of cells in the G0/G1 phase, and increased the proportion of cells in G2/M. Human and mouse embryonic stem cell (ESC) subpopulations display heterogeneous expression of IK_DR_ currents^43^. In mouse ESCs, depolarization by blockade of Kv channels results in not only decreased proliferation, but also loss of pluripotency, as measured by loss of Oct-4, Sox-2, and Nanog expression, and an increase in expression of early germ layer markers, particularly of the mesendoderm and trophectoderm^44^. The state of V_mem_ polarization may thus regulate the differentiation capacity of ESCs. Since Kv channel activity is largely responsible for setting the resting membrane potential in these cells, Kv activity may function as a switch between the decision to self-renew or to differentiate^44^.

In cells from the adult mouse hippocampus, V_mem_ depolarization was found to activate stem-like properties in latent stem and progenitor cells, similar to the role of resting potential in directing embryonic brain precursor cells^45^. Depolarization with KCl increased the number of neurospheres generated from hippocampal cells and activated a subpopulation of stem and progenitor cells that had greater self-renewal capacity than unactivated cells, that maintained multipotentiality, and that had the capacity to generate neurons^46^. In bone marrow-derived hMSCs, ionic currents including IK_Ca_, IK_DR_, transient outward K^+^ currents, and slow-activating currents have been detected. Expression of Kv4.2, Kv4.3, MaxiK, L-type Ca^2+^ channels, and HCN ion channels may be responsible for the detected currents^33^,^34^. V_mem_ hyperpolarization has been shown to accompany and be required for differentiation of hMSCs toward osteogenic and adipogenic lineages. Disruption of normal V_mem_ progression by induced depolarization (via ouabain blockade of Na^+^/K^+^ ATPase activity or by high extracellular [K^+^]) resulted in reduced differentiation capacity, while hyperpolarization by K^+^ channel agonists resulted in augmented osteogenic differentiation. These results suggest that V_mem_ can be a tractable control point for modulation of stem cell differentiation in a bi-directional fashion^35^. Indeed, *in vivo*, rational control of V_mem_ can induce organ-level reprogramming, inducing whole eyes and brains in ectopic locations^47^,^48^.

Although these and other studies suggest that the electrophysiological state can be modulated for desired functions, a major concern in employing such manipulations for stem cell therapies and tissue engineering is the issue of stem cell heterogeneity, especially related to differentiation capacity and differentiation lineage biases. The donor-to-donor heterogeneity of hMSCs is well documented. In a study of bone marrow stromal cells from seventeen donors, cell growth rate exhibited a 12-fold variation^17^. Colony-forming potential and cell surface marker expression also differ between donors^20^,^21^. A proteomic profiling study revealed significant donor variation in protein expression in undifferentiated hMSCs across six donors^22^. Of the total number of non-redundant proteins from the six cell lines, one out of six identified proteins was expressed in only one cell line and not the others, and only 13% of the total identified proteins was expressed in all cell lines^22^. Differentiation potential is also a major phenotypic variable among hMSCs. For example, ALPL enzyme activity varied up to 40-fold in undifferentiated hMSCs from seventeen donors, and the level of ALPL induction upon osteogenic stimulation varied between 1- and 17-fold^17^. A separate study comparing nineteen donors found a similar variability in ALPL expression: the percentage of ALPL-expressing cells in a non-induced population varied between 1 and 33%, and the level of ALPL induction upon stimulation varied between 1.3- and 3.8-fold^18^. Osteogenic gene expression was also studied in multiple hMSC donors, and investigators found wide variations in the mRNA levels of *ALPL*, *IBSP*, parathyroid hormone receptor (pTHR), collagen type I, osteocalcin, osteopontin, osteonectin, and S100A4^17^,^18^. Additionally, two out of three donors’ hMSCs exhibited *in vitro* calcium deposition, and three out of four donors’ hMSCs induced bone formation in *in vivo* cell transplantation experiments^18^. In a separate study, *in vivo* bone forming capacity was reported in 58.8% of strains from single colonies derived from four donors^19^. A study comparing the adipogenic potential of hMSCs from two donors reported differences in adipocyte precursor frequency and adipogenic gene expression changes over the course of passaging^20^. Another study quantified endothelial-like differentiation of hMSCs from twenty donors, and large variability was found in the cells’ endothelial-like tube formation efficiency and endothelial gene expression^23^. Collectively, these data point to significant heterogeneity in the inherent properties of hMSCs from different donors, particularly in their differentiation capacity.

Data from the present study is largely in agreement with the reports of inter-donor hMSC heterogeneity in the literature. Of all the osteogenic markers examined in our study, ALPL expression and activity were the most variable among donors, regardless of the state of membrane polarization. ALPL activity varied by as much as two orders of magnitude between donors (Supplemental Fig. 1). Induction of *ALPL* expression by osteogenic stimulation differed by as much as one order of magnitude between donors (Fig. 1). In some donors, osteogenic induction did not cause any increase in ALPL activity or expression (Fig. 1A–E, Supplemental Fig. 1) compared to undifferentiated controls, potentially calling into question whether ALPL can be considered an osteogenic marker. Some donors that failed to exhibit increases in ALPL activity or expression nevertheless exhibited expected patterns for other osteogenic markers (e.g., *IBSP* expression or calcium content). *IBSP* expression and calcium content were consistently and significantly upregulated upon osteogenic induction in all donor cells. Thus, these data suggest that assessment of osteogenic differentiation should ideally be performed with more than one osteogenic marker, and that the markers should be validated in each donor’s cells.

For adipogenic differentiation, we found that the transcripts chosen for evaluation generally performed well as adipogenic markers (exhibited significant increases upon adipogenic induction). Transcript expression was consistently responsive to adipogenic induction across donors. One donor showed a small variation, where due to large variability within the population, the upregulation of PPARG to adipogenic induction was not statistically significant (Donor E, Fig. 4E). Oil Red O staining was also a strong assay for detecting lipid accumulation in induced cells compared to undifferentiated cells (e.g., Fig. 7, compare AD column with CON column). We observed significant inter-donor variation in the degree to which each donor’s cells upregulated adipogenic transcript expression compared to their respective undifferentiated cells. For example, the level of *LPL* induction varied by up to 4-fold between donors (Fig. 5), and the level of *FABP4* induction varied approximately up to 14-fold between donors (Fig. 6). These differences are reflected in the differences in frequency of Oil Red O stained cells between donors (Fig. 7, compare images in AD column). These data indicate that there was substantial variation in the degree to which each donor’s hMSCs could express each marker, potentially reflecting differences in adipogenic capacity between donors.

Using the tissue-specific markers that performed adequately as differentiation markers (i.e., *IBSP*, calcium, *PPARG*, *LPL*, *FABP4*, and Oil Red O), we then examined whether different donors’ cells responded consistently to V_mem_ depolarization. In evaluating whether these markers were consistently depolarization-responsive, we chose to focus on quantifying the fraction of donors that exhibited statistically significant changes upon depolarization, rather than the magnitude of the change (e.g., fold-change values). Due to the inherent variation in each donor’s expression of differentiation markers, the fold-changes induced by depolarization are only meaningful when considered in the context of the cells’ baseline differentiation capacity. Additionally, because differentiation is a complex process involving multiple cell functions, it is important to evaluate a set of characteristic markers rather than the fold-change magnitude of one marker alone. Therefore, in determining which markers responded most consistently across donors, we did not define specific criteria for the magnitude of gene expression changes. Rather, we looked for consistency in the response of these markers (upregulation or downregulation) across multiple donors.

HK-induced suppression of osteogenic differentiation was seen in three out of five donors as measured by *IBSP* expression, and five out of five donors as measured by calcium content (Figs 2A–E and 3A–E). When osteogenic cells were depolarized after pre-differentiation, four out of five donors exhibited lowered *IBSP* expression and four out of five donors exhibited lower calcium content compared to non-depolarized cells in four out of five donors. Interestingly, the donor that responded atypically was different for *IBSP* and calcium in this latter study, indicating that heterogeneity can be seen in the response of individual differentiation markers, rather than the entire osteogenic profile.

During adipogenic differentiation with HK, *LPL* suppression was observed in all five donors, while *PPARG* and *FABP4* suppression were observed in four out of five donors. The atypical response for *PPARG* and *FABP4* were both found in Donor E, where HK upregulated transcript expression instead of suppressing it. Evaluation of lipid accumulation by Oil Red O staining also revealed that Donor E cells did not exhibit an appreciable difference in staining between cells differentiated with or without HK. Thus, in contrast to osteogenic differentiation, the outlier donor for adipogenic differentiation was the same donor for all markers. After adipogenic pre-differentiation, HK-induced suppression was observed in three out of five donors for *PPARG* expression, and five out of five donors for both *LPL* and *FABP4* expression. It is worth noting that *PPARG* is considered an early adipogenic marker, while *LPL* and *FABP4* are markers for later stages of differentiation. This could explain why *PPARG* is less responsive to late depolarization in some donors: in these cells, perhaps the window of time for significant *PPARG* modulation has passed, and the cell instead modulates expression of the markers that are more relevant to the adipogenic stage at which HK is introduced (e.g., *LPL* and *FABP4*).

The present study focuses exclusively on inter-donor stem cell heterogeneity. However, additional studies in the literature have reported not only inter-donor stem cell variability, but also variability within a stem cell population from a single donor source. *In vitro* differentiation studies of bone marrow-derived hMSCs have shown that this population consists of a mixture of undifferentiated stem/progenitor cells and lineage-restricted precursors which have different capacities for differentiation toward osteogenic, adipogenic, and chondrogenic lineages^17^,^19^,^49^,^50^. Other adult stem cell populations exhibiting heterogeneity include intestinal stem cells and hematopoietic stem cells^51^. Similarly, cell populations differentiated from embryonic stem cells display heterogeneous phenotypes despite stimulation with specific growth factors^52^,^53^,^54^. Electrophysiological characterization of the phenotypically-distinct subpopulations within these stem cell populations may provide clues about how ion channels and currents contribute to the differences in their differentiation capacities and lineage biases, as has been shown in neuroblastoma cells^55^. Another source of heterogeneity may be the specific organ or anatomical location from which the cells are derived. Studies that compared mesenchymal stem cells derived from the bone marrow, adipose tissue, placenta, and umbilical cord^56^,^57^,^58^ reported differences in their proliferative and differentiation capacities. Similarly, fetal and adult human fibroblasts derived from skin at different anatomical locations displayed different characteristic gene expression patterns^59^. While the data in the present study do not provide specific information about intra-population variability in hMSCs or about variability arising from different tissue sources, they do suggest that the assays that performed most consistently across donors may be the most useful for future studies addressing other sources of variability in hMSCs.

Future studies may also examine whether three-dimensional (3D) cultures of hMSCs would exhibit similar levels of heterogeneity during depolarization as the 2D cultures in this study. We previously developed a 3D culture system to study the effect of membrane potential stimulation on wound healing^60^. Interestingly, high K^+^ treatment had different effects depending on spatial location within the wound model (wound center vs. surrounding tissue). One potential explanation for the different observed responses is that the spatial variables introduced by the 3D culture system may intensify any intra-population differences. From these observations, if 3D models were used to compare different donors’ cells, we would expect that in addition to seeing variability among different donors, as in the current study, we would also observe increased intra-population variability within a single 3D culture system caused by spatial effects.

Overall, this study provided an analysis of hMSC heterogeneity across multiple donor sources, specifically with respect to their inherent differentiation potential and their differentiation response to electrophysiological perturbations. Our results indicate that, consistent with the literature, there was a baseline degree of variability among the donor cells’ ability to express tissue-specific markers. The specifics of this heterogeneity cannot always be summarized by identifying one outlier donor or one outlier assay, suggesting the need to systematically characterize each cell line that will be used for further analysis. Furthermore, there was additional variability in donor cell response to V_mem_ depolarization, and that this variability was not always predictable based on the inherent heterogeneity of hMSC differentiation capacity. However, for each donor, there was at least one osteogenic marker and one adipogenic marker that responded to depolarization in the expected manner. Based on this screen, we suggest that *IBSP* expression, calcium deposition, *LPL* expression, and *FABP4* expression are the preferred markers for assessing differentiation response to V_mem_ depolarization, but a full characterization of a cell source with all markers is preferred. Donor-specific characterization of primary stem cells will be increasingly important as stem cell transplantation and personalized medicine strategies are being developed for clinical therapies.

## Additional Information

**How to cite this article**: Sundelacruz, S. *et al.* Comparison of the depolarization response of human mesenchymal stem cells from different donors. *Sci. Rep.* **5**, 18279; doi: 10.1038/srep18279 (2015).

## Supplementary Material

Supplementary Information

## References

[b1] Friedenstein A. J., Petrakova K. V., Kurolesova A. I. & Frolova G. P. Heterotopic of bone marrow. Analysis of precursor cells for osteogenic and hematopoietic tissues. Transplantation 6, 230–247 (1968). 5654088

[b2] Friedenstein A. J., Piatetzky S. II & Petrakova K. V. Osteogenesis in transplants of bone marrow cells. J Embryol Exp Morphol 16, 381–390 (1966). 5336210

[b3] Boregowda S. V. & Phinney D. G. Therapeutic applications of mesenchymal stem cells: current outlook. BioDrugs 26, 201–208, 10.2165/11632790-000000000-00000 (2012). 22708790

[b4] Pacini S. Deterministic and stochastic approaches in the clinical application of mesenchymal stromal cells (MSCs). Front Cell Dev Biol 2, 50, 10.3389/fcell.2014.00050 (2014). 25364757 PMC4206995

[b5] Phinney D. G. Functional heterogeneity of mesenchymal stem cells: implications for cell therapy. J Cell Biochem 113, 2806–2812, 10.1002/jcb.24166 (2012). 22511358

[b6] Das R., Jahr H., van Osch G. J. & Farrell E. The role of hypoxia in bone marrow-derived mesenchymal stem cells: considerations for regenerative medicine approaches. Tissue Eng Part B Rev 16, 159–168, 10.1089/ten.TEB.2009.0296 (2010). 19698058

[b7] Guilak F. *et al.* Control of stem cell fate by physical interactions with the extracellular matrix. Cell Stem Cell 5, 17–26, 10.1016/j.stem.2009.06.016 (2009). 19570510 PMC2768283

[b8] Hronik-Tupaj M. & Kaplan D. L. A review of the responses of two- and three-dimensional engineered tissues to electric fields. Tissue Eng Part B Rev 18, 167–180, 10.1089/ten.TEB.2011.0244 (2012). 22046979 PMC3357076

[b9] Kolf C. M., Cho E. & Tuan R. S. Mesenchymal stromal cells. Biology of adult mesenchymal stem cells: regulation of niche, self-renewal and differentiation. Arthritis Res Ther 9, 204, 10.1186/ar2116 (2007). 17316462 PMC1860068

[b10] Rehfeldt F., Engler A. J., Eckhardt A., Ahmed F. & Discher D. E. Cell responses to the mechanochemical microenvironment–implications for regenerative medicine and drug delivery. Adv Drug Deliv Rev 59, 1329–1339, 10.1016/j.addr.2007.08.007 (2007). 17900747 PMC4124491

[b11] Brooke G. *et al.* Therapeutic applications of mesenchymal stromal cells. Semin Cell Dev Biol 18, 846–858, 10.1016/j.semcdb.2007.09.012 (2007). 18024097

[b12] Le Blanc K. & Ringden O. Immunomodulation by mesenchymal stem cells and clinical experience. J Intern Med 262, 509–525, 10.1111/j.1365-2796.2007.01844.x (2007). 17949362

[b13] Salem H. K. & Thiemermann C. Mesenchymal stromal cells: current understanding and clinical status. Stem Cells 28, 585–596, 10.1002/stem.269 (2010). 19967788 PMC2962904

[b14] Sato K., Ozaki K., Mori M., Muroi K. & Ozawa K. Mesenchymal stromal cells for graft-versus-host disease: basic aspects and clinical outcomes. J Clin Exp Hematop 50, 79–89 (2010). 21123965 10.3960/jslrt.50.79

[b15] Bianco P. *et al.* The meaning, the sense and the significance: translating the science of mesenchymal stem cells into medicine. Nat Med 19, 35–42, 10.1038/nm.3028 (2013). 23296015 PMC3998103

[b16] Phinney D. G. Building a consensus regarding the nature and origin of mesenchymal stem cells. J Cell Biochem Suppl 38, 7–12 (2002). 12046852 10.1002/jcb.10084

[b17] Phinney D. G. *et al.* Donor variation in the growth properties and osteogenic potential of human marrow stromal cells. J Cell Biochem 75, 424–436 (1999). 10536366

[b18] Siddappa R., Licht R., van Blitterswijk C. & de Boer J. Donor variation and loss of multipotency during *in vitro* expansion of human mesenchymal stem cells for bone tissue engineering. J Orthop Res 25, 1029–1041, 10.1002/jor.20402 (2007). 17469183

[b19] Kuznetsov S. A. *et al.* Single-colony derived strains of human marrow stromal fibroblasts form bone after transplantation *in vivo*. J Bone Miner Res 12, 1335–1347, 10.1359/jbmr.1997.12.9.1335 (1997). 9286749

[b20] Lo Surdo J. & Bauer S. R. Quantitative approaches to detect donor and passage differences in adipogenic potential and clonogenicity in human bone marrow-derived mesenchymal stem cells. Tissue Eng Part C Methods 18, 877–889, 10.1089/ten.TEC.2011.0736 (2012). 22563812 PMC3483050

[b21] Siegel G. *et al.* Phenotype, donor age and gender affect function of human bone marrow-derived mesenchymal stromal cells. BMC Med 11, 146, 10.1186/1741-7015-11-146 (2013). 23758701 PMC3694028

[b22] Mindaye S. T., Ra M., Lo Surdo J. L., Bauer S. R. & Alterman M. A. Global proteomic signature of undifferentiated human bone marrow stromal cells: evidence for donor-to-donor proteome heterogeneity. Stem Cell Res 11, 793–805, 10.1016/j.scr.2013.05.006 (2013). 23792435

[b23] Portalska K. J. *et al.* The effect of donor variation and senescence on endothelial differentiation of human mesenchymal stromal cells. Tissue Eng Part A 19, 2318–2329, 10.1089/ten.TEA.2012.0646 (2013). 23676150

[b24] Kretlow J. D. *et al.* Donor age and cell passage affects differentiation potential of murine bone marrow-derived stem cells. BMC Cell Biol 9, 60, 10.1186/1471-2121-9-60 (2008). 18957087 PMC2584028

[b25] Blackiston D. J., McLaughlin K. A. & Levin M. Bioelectric controls of cell proliferation: ion channels, membrane voltage and the cell cycle. Cell Cycle 8, 3527–3536 (2009). 19823012 10.4161/cc.8.21.9888 PMC2862582

[b26] Levin M. Molecular bioelectricity: how endogenous voltage potentials control cell behavior and instruct pattern regulation *in vivo*. Mol Biol Cell 25, 3835–3850, 10.1091/mbc.E13-12-0708 (2014). 25425556 PMC4244194

[b27] Levin M. Endogenous bioelectrical networks store non-genetic patterning information during development and regeneration. J Physiol 592, 2295–2305, 10.1113/jphysiol.2014.271940 (2014). 24882814 PMC4048089

[b28] Levin M. & Stevenson C. G. Regulation of cell behavior and tissue patterning by bioelectrical signals: challenges and opportunities for biomedical engineering. Annu Rev Biomed Eng 14, 295–323, 10.1146/annurev-bioeng-071811-150114 (2012). 22809139 PMC10472538

[b29] Pillozzi S. & Becchetti A. Ion channels in hematopoietic and mesenchymal stem cells. Stem Cells Int 2012, 217910, 10.1155/2012/217910 (2012). 22919401 PMC3420091

[b30] Sundelacruz S., Levin M. & Kaplan D. L. Role of membrane potential in the regulation of cell proliferation and differentiation. Stem Cell Rev 5, 231–246, 10.1007/s12015-009-9080-2 (2009). 19562527 PMC10467564

[b31] Tai G., Reid B., Cao L. & Zhao M. Electrotaxis and wound healing: experimental methods to study electric fields as a directional signal for cell migration. Methods Mol Biol 571, 77–97, 10.1007/978-1-60761-198-1_5 (2009). 19763960

[b32] Levin M. Molecular bioelectricity: how endogenous voltage potentials control cell behavior and instruct pattern regulation *in vivo*. Mol Biol Cell 25, 3835–3850, 10.1091/mbc.E13-12-0708 (2014). 25425556 PMC4244194

[b33] Heubach J. F. *et al.* Electrophysiological properties of human mesenchymal stem cells. J Physiol 554, 659–672 (2004). 14578475 10.1113/jphysiol.2003.055806 PMC1664789

[b34] Li G. R., Sun H., Deng X. & Lau C. P. Characterization of ionic currents in human mesenchymal stem cells from bone marrow. Stem Cells 23, 371–382 (2005). 15749932 10.1634/stemcells.2004-0213

[b35] Sundelacruz S., Levin M. & Kaplan D. L. Membrane potential controls adipogenic and osteogenic differentiation of mesenchymal stem cells. PLoS ONE 3, e3737 (2008). 19011685 10.1371/journal.pone.0003737 PMC2581599

[b36] Sundelacruz S., Levin M. & Kaplan D. L. Depolarization alters phenotype, maintains plasticity of predifferentiated mesenchymal stem cells. Tissue Eng Part A 19, 1889–1908, 10.1089/ten.tea.2012.0425.rev (2013). 23738690 PMC3726227

[b37] Mustard J. & Levin M. Bioelectrical Mechanisms for Programming Growth and Form: Taming Physiological Networks for Soft Body Robotics. Soft Robotics 1, 169–191, 10.1089/soro.2014.0011 (2014).

[b38] Tseng A. & Levin M. Cracking the bioelectric code: Probing endogenous ionic controls of pattern formation. Commun Integr Biol 6, 1–8 (2013). 10.4161/cib.22595 PMC3689572 23802040

[b39] Levin M. Reprogramming cells and tissue patterning via bioelectrical pathways: molecular mechanisms and biomedical opportunities. Wiley Interdiscip Rev Syst Biol Med 5, 657–676, 10.1002/wsbm.1236 (2013). 23897652 PMC3841289

[b40] Funk R. H., Monsees T. & Ozkucur N. Electromagnetic effects - From cell biology to medicine. Prog Histochem Cytochem 43, 177–264 (2009). 19167986 10.1016/j.proghi.2008.07.001

[b41] Zhao M. *et al.* Electrical signaling in control of ocular cell behaviors. Prog Retin Eye Res 31, 65–88, 10.1016/j.preteyeres.2011.10.001 (2012). 22020127 PMC3242826

[b42] Wang E. T. & Zhao M. Regulation of tissue repair and regeneration by electric fields. Chin J Traumatol 13, 55–61 (2010). 20109370

[b43] Wang K. *et al.* Electrophysiological properties of pluripotent human and mouse embryonic stem cells. Stem Cells 23, 1526–1534 (2005). 16091557 10.1634/stemcells.2004-0299

[b44] Ng S. Y. *et al.* Role of voltage-gated potassium channels in the fate determination of embryonic stem cells. J Cell Physiol 224, 165–177 (2010). 20333647 10.1002/jcp.22113

[b45] Lange C. *et al.* The H(+) vacuolar ATPase maintains neural stem cells in the developing mouse cortex. Stem Cells Dev 20, 843–850, 10.1089/scd.2010.0484 (2011). 21126173 PMC3128780

[b46] Walker T. L. *et al.* Latent stem and progenitor cells in the hippocampus are activated by neural excitation. J Neurosci 28, 5240–5247 (2008). 18480280 10.1523/JNEUROSCI.0344-08.2008 PMC6670644

[b47] Pai V. P., Aw S., Shomrat T., Lemire J. M. & Levin M. Transmembrane voltage potential controls embryonic eye patterning in Xenopus laevis. Development 139, 313–323, 10.1242/dev.073759 (2012). 22159581 PMC3243095

[b48] Beane W. S., Morokuma J., Adams D. S. & Levin M. A Chemical genetics approach reveals H,K-ATPase-mediated membrane voltage is required for planarian head regeneration. Chem Biol 18, 77–89 (2011). 21276941 10.1016/j.chembiol.2010.11.012 PMC3278711

[b49] Muraglia A., Cancedda R. & Quarto R. Clonal mesenchymal progenitors from human bone marrow differentiate *in vitro* according to a hierarchical model. J Cell Sci 113(Pt 7), 1161–1166 (2000). 10704367 10.1242/jcs.113.7.1161

[b50] Russell K. C. *et al.* *In vitro* high-capacity assay to quantify the clonal heterogeneity in trilineage potential of mesenchymal stem cells reveals a complex hierarchy of lineage commitment. Stem Cells 28, 788–798, 10.1002/stem.312 (2010). 20127798

[b51] Graf T. & Stadtfeld M. Heterogeneity of Embryonic and Adult Stem Cells. Cell Stem Cell 3, 480–483 (2008). 18983963 10.1016/j.stem.2008.10.007

[b52] Odorico J. S., Kaufman D. S. & Thomson J. A. Multilineage differentiation from human embryonic stem cell lines. Stem Cells 19, 193–204 (2001). 11359944 10.1634/stemcells.19-3-193

[b53] Schuldiner M., Yanuka O., Itskovitz-Eldor J., Melton D. A. & Benvenisty N. Effects of eight growth factors on the differentiation of cells derived from human embryonic stem cells. Proc Natl Acad Sci USA 97, 11307–11312 (2000). 11027332 10.1073/pnas.97.21.11307 PMC17196

[b54] Shamblott M. J. *et al.* Human embryonic germ cell derivatives express a broad range of developmentally distinct markers and proliferate extensively *in vitro*. Proc Natl Acad Sci USA 98, 113–118 (2001). 11134532 10.1073/pnas.021537998 PMC14553

[b55] Biagiotti T. *et al.* Cell renewing in neuroblastoma: electrophysiological and immunocytochemical characterization of stem cells and derivatives. Stem Cells 24, 443–453, 10.1634/stemcells.2004-0264 (2006). 16100002

[b56] Baksh D., Yao R. & Tuan R. S. Comparison of proliferative and multilineage differentiation potential of human mesenchymal stem cells derived from umbilical cord and bone marrow. Stem Cells 25, 1384–1392 (2007). 17332507 10.1634/stemcells.2006-0709

[b57] Barlow S. *et al.* Comparison of human placenta- and bone marrow-derived multipotent mesenchymal stem cells. Stem Cells Dev 17, 1095–1107 (2008). 19006451 10.1089/scd.2007.0154

[b58] Kern S., Eichler H., Stoeve J., Klüter H. & Bieback K. Comparative analysis of mesenchymal stem cells from bone marrow, umbilical cord blood, or adipose tissue. Stem Cells 24, 1294–1301 (2006). 16410387 10.1634/stemcells.2005-0342

[b59] Chang H. Y. *et al.* Diversity, topographic differentiation, and positional memory in human fibroblasts. Proc Natl Acad Sci USA 99, 12877–12882, 10.1073/pnas.162488599 (2002). 12297622 PMC130553

[b60] Sundelacruz S., Li C., Choi Y. J., Levin M. & Kaplan D. L. Bioelectric modulation of wound healing in a 3D *in vitro* model of tissue-engineered bone. Biomaterials 34, 6695–6705, 10.1016/j.biomaterials.2013.05.040 (2013). 23764116 PMC3724996

