# Supplementary material for: Comparison of the depolarization response of human mesenchymal stem cells from different donors
Source: Sci Rep. 2015 Dec 14;5:18279. doi: 10.1038/srep18279 (PMC4677319; doi:10.1038/srep18279)
Supplement: Supplementary Information [file srep18279-s1.pdf]

Comparison of the depolarization response of human mesenchymal stem cells from different donors

Sarah Sundelacruz[1], Michael Levin[2], David L. Kaplan*[1]

[1] Department of Biomedical Engineering, Tufts University, Medford, MA, USA
[2] Department of Biology, and Center for Regenerative and Developmental Biology, Tufts University, Medford, MA, USA

Sarah Sundelacruz
4 Colby Street
Tufts University
Medford, MA 02155 USA
1 617 627 2670 (phone)
1 617 627 3231 (fax)
Sarah.Sundelacruz@tufts.edu

Michael Levin
Suite 4600
200 Boston Avenue
Tufts University
Medford, MA 02155 USA
1 617 627 6161 (phone)
1 617 627 6121 (fax)
Michael.Levin@tufts.edu

David L. Kaplan (*corresponding author)
4 Colby Street
Tufts University
Medford, MA 02155 USA
1 617 627 3251 (phone)
1 617 627 3231 (fax)
David.Kaplan@tufts.edu

Supplemental Figures

Supplemental Figure 1

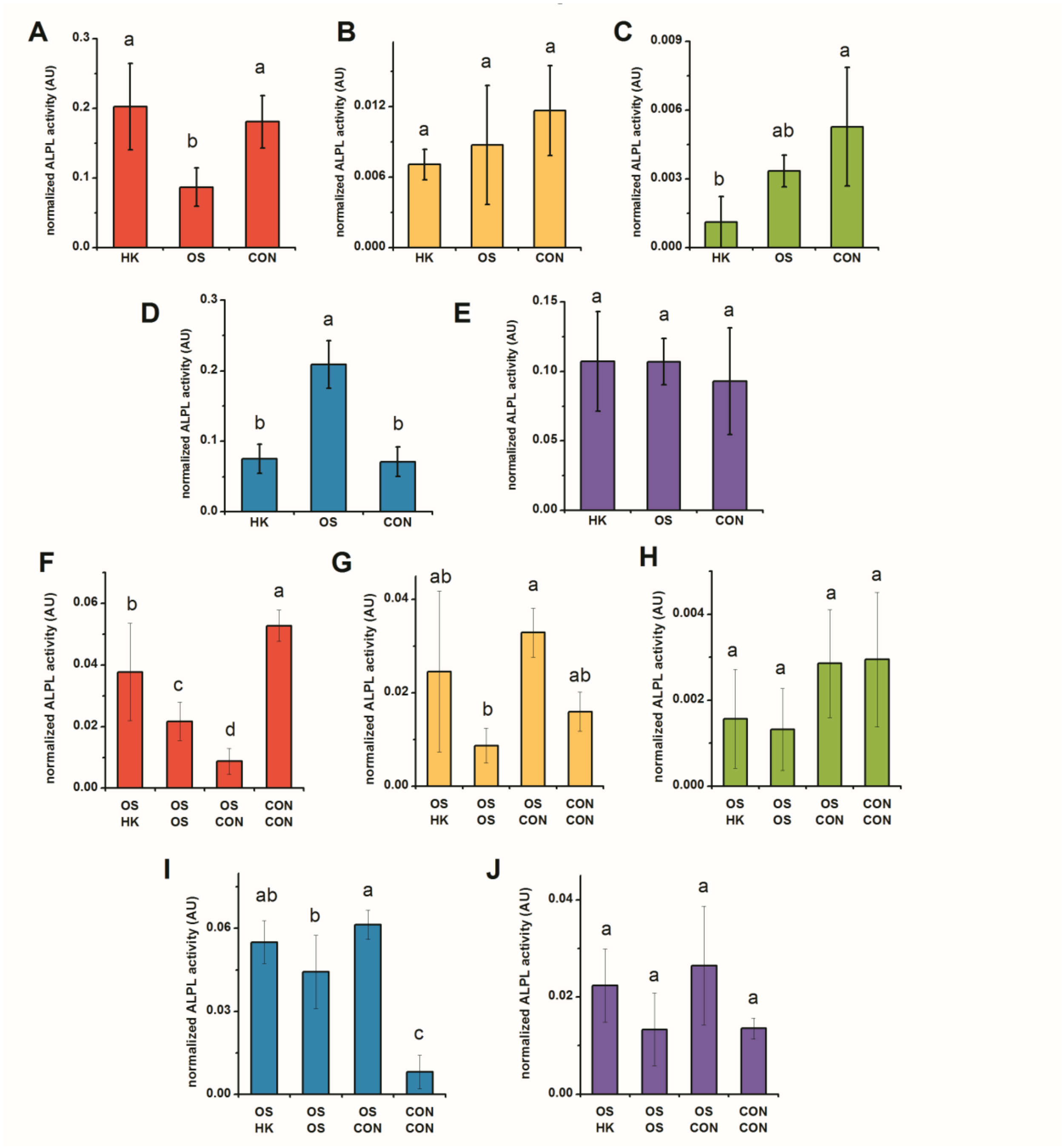

Supplemental Figure 2

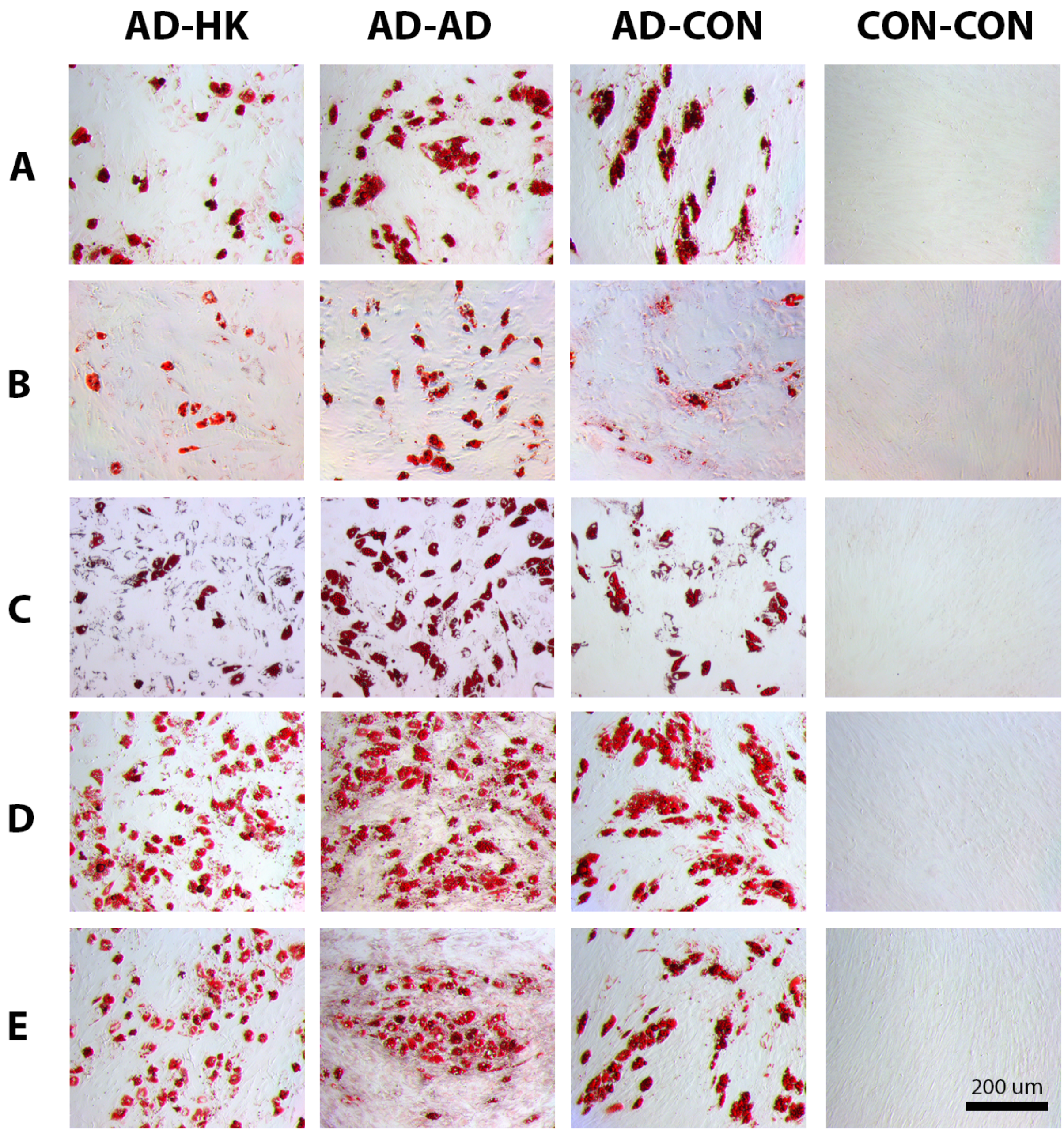

Supplemental Figure Legends

Supplemental Figure 1. ALPL enzymatic activity in hMSCs depolarized during osteogenic differentiation (A-E) and in pre-differentiated hMSCs (F-J).

(A-E) ALPL activity was quantified by an enzymatic assay after 14 days of hMSC culture in osteogenic medium + 40 mM K^+ (HK), osteogenic medium (OS), or control medium (CON). (F-J) hMSCs were pre-treated for 2 weeks (osteogenic (OS) or control medium (CON)) before switching to osteogenic medium + 40 mM K^+ (HK), osteogenic medium (OS), or control medium (CON) for 1 week. ALPL activity was quantified by an enzymatic assay after the entire 3 weeks of culture. Data points represent mean normalized enzyme activity (AU) ± standard deviation, n = 3-8. Panels A and F; B and G; C and H; D and I; and E and J correspond to data from Donors A, B, C, D, and E, respectively. Different letters over bar graphs represent statistically different groups as determined by one-way ANOVA and the Tukey-Kramer post-hoc test, $p < 0.05$.

Supplemental Figure 2. Oil Red O staining of lipids in hMSCs depolarized after adipogenic pre-differentiation.

hMSCs were pre-treated for 2 weeks (adipogenic (AD) or control medium (CON)) before switching to adipogenic medium + 80 mM K^+ (HK), adipogenic medium (AD), or control medium (CON) for 1 week. Oil Red O staining was performed after the entire 3 weeks of culture. Rows A, B, C, D, and E correspond to images from Donors A, B, C, D, and E, respectively. Scale bar = 200 µm.
